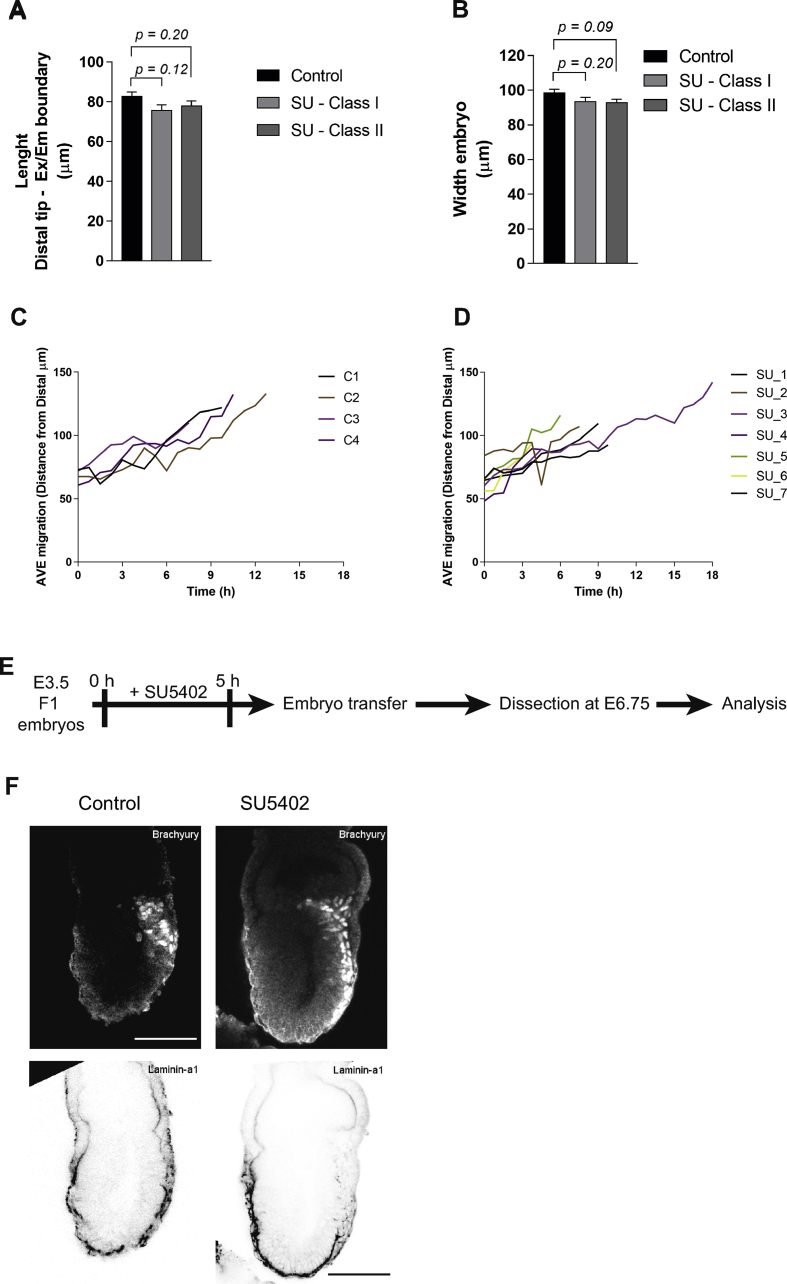# Concerted cell divisions in embryonic visceral endoderm guide anterior visceral endoderm migration

**DOI:** 10.1016/j.ydbio.2019.03.016

**Published:** 2019-06-15

**Authors:** Francesco Antonica, Lorenzo Carlo Orietti, Richard Lester Mort, Magdalena Zernicka-Goetz

**Affiliations:** a Mammalian Embryo and Stem Cell Group, University of Cambridge, Department of Physiology, Development and Neuroscience, Downing Street, Cambridge, CB2 3DY, UK; b Division of Biomedical and Life Sciences, Faculty of Health and Medicine, Lancaster University, Bailrigg, Furness Building, Lancaster LA1 4YG, UK

**Keywords:** 4D live imaging, Mouse embryo, Post-implantation morphogenesis, Visceral endoderm

## Abstract

Migration of Anterior Visceral Endoderm (AVE) is a critical symmetry breaking event in the early post-implantation embryo development and is essential for establishing the correct body plan. Despite much effort, cellular and molecular events influencing AVE migration are only partially understood. Here, using time-lapse live imaging of mouse embryos, we demonstrate that cell division in the embryonic visceral endoderm is coordinated with AVE migration. Moreover, we demonstrate that temporal inhibition of FGF signalling during the pre-implantation specification of embryonic visceral endoderm perturbs cell cycle progression, thus affecting AVE migration. These findings demonstrate that coordinated cell cycle progression during the implantation stages of development is important for post-implantation morphogenesis in the mouse embryo.

## Highlights

• Cell divisions are concerted in embryonic visceral endoderm of post-implantation mouse embryos.
• AVE migration is dependent on concerted cell divisions.
• FGF signalling inhibition during PE specification affects coordinated mitosis and AVE migration in post-implantation embryos.

## 1 Introduction

The transition from pre-to post-implantation in mouse embryogenesis is marked by remarkable morphological changes; the round implanting blastocyst is transformed into an elongated egg cylinder with an Anterior-Posterior (AP) axis. During implantation, the polar trophectoderm of the blastocyst transforms into the extra-embryonic ectoderm (ExE) which signals posterior development to the epiblast (EPI). The primitive endoderm (PE) of the blastocyst differentiates into parietal endoderm and the visceral endoderm (VE), which constitutes the outer epithelial layer that surrounds the ExE and EPI. By embryonic day (E)5.5, a Nodal gradient becomes established in the EPI leading to the emergence of the proximal-distal axis (Arnold and Robertson, 2009). This signalling is distally inhibited by the Nodal antagonists Lefty1 and Cerberus (Cerl), secreted by a specialised subpopulation of embryonic VE cells known as the distal visceral endoderm (DVE) (Belo et al., 1997; Yamamoto et al., 2004). A few hours after their specification, DVE cells acquire a migratory morphology and together with the newly formed AVE (Takaoka et al., 2011; Morris et al., 2012) move unilaterally towards the embryonic/extra-embryonic (Em/Ex) boundary (Srinivas et al., 2004), the future anterior side of the embryo. The localization of AVE on one side of the embryo will mark the future anterior side and the primitive streak will form on the posterior side (Brennan et al., 2001). The cellular mechanisms that play a role in the asymmetric positioning of the AVE are not well understood (Stower and Srinivas, 2014). Previous studies have revealed that AVE cells actively migrate and change their shape through remodelling of the cytoskeleton, which results in cell intercalation (Migeotte et al., 2010; Morris et al., 2012; Omelchenko et al., 2014; Srinivas et al., 2004; Stower and Srinivas, 2014; Trichas et al., 2012). However, how AVE migration relates to cell cycle progression in the VE has been a matter of debate (Stuckey et al., 2011a; Yamamoto et al., 2004). Indeed, Stuckey and colleagues did not observe any difference in the frequency of mitoses within VE by analysing fixed samples stained for mitotic markers. They also proposed that cell proliferation in the EPI and its subsequent growth and expansion lead to the migration of AVE (Stuckey et al., 2011a). By contrast, Yamamoto and colleagues proposed that AVE migration could be driven by differential proliferation within the VE resulting from asymmetry in Nodal signalling (Yamamoto et al., 2004). Both studies were based on analyses of fixed samples and none have analysed the dynamics of cell divisions during AVE migration and whether there is any spatio-temporal coordination between the two events. To address this, we use time-lapse imaging of visceral endoderm cells in a cell cycle reporter mouse strain and show that AVE migration is coordinated with progression through the cell cycle. Moreover, our results suggest that coordinated cell cycle progression originates from signalling events in pre-implantation development.

## 2 Results

### 2.1 Spatio-temporal correlation between AVE migration and coordinated cell division

As AVE migration commences around E5.5, we focused on analysing the dynamics of cell cycle progression around this time. In order to relate cell cycle progression to AVE migration, we first generated double transgenic embryos for the AVE marker Cerl (Cerl-GFP) (Mesnard et al., 2004) and the cell cycle reporter R26Fucci2a (Fluorescent Ubiquitination-based Cell Cycle Indicator) (Mort et al., 2014) (Fig. 1A). The bicistronic Fucci2a construct encodes truncated versions of the Cdt1 and Geminin proteins that are degraded reciprocally during cell cycle progression; mCherry-hCdt1(30/120) levels peak in G1, and mVenus-hGem(1/110) levels peak in S/G2/M phases. Fucci2a thus allows assessment of cell cycle progression using live imaging (Mort et al., 2014) (Fig. 1A). We were able to distinguish GFP and mVenus signals and thereby identify Cerl-expressing cells and cells in S/G2/M (Fig. S1A). We assessed cell cycle progression approximately 12 h and 24 h after implantation by recovering embryos at E5.0 and E5.5, respectively. R26Fucci2a/CerI-GFP or R26Fucci2a embryos recovered at E5.5 or E5.0 respectively and imaged with multi-photon confocal microscopy both showed mVenus-expressing cells (in S/G2/M phase) (Fig. 1B, upper and middle panels; Fig. 2E). However, we could not detect mCherry labelled cells, most likely due to the short G1 phase characteristic of early post-implantation development, a similar finding to one described for mouse embryonic stem cells (Mort et al., 2014). Indeed, we were only able to detect a few positive mCherry labelled cells in the distal part of late E6.5 embryos when we applied higher laser power, incompatible with live imaging experiments (Fig. S1B). This is in line with previous findings that it is rare to detect G1 cells in Fucci embryos between E5.5 and E6.5 (Abe et al., 2013).

**Fig. 1:**
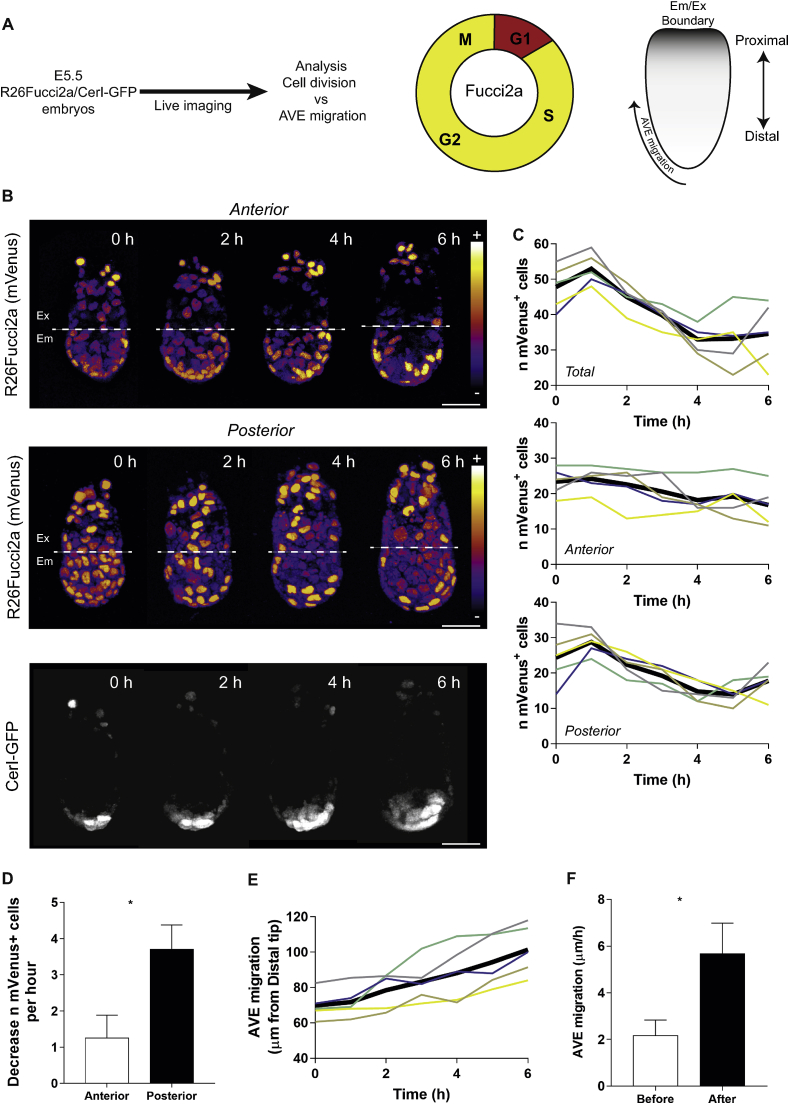
**Concerted cell divisions occur during AVE migration (**A) Schematic view of the experimental design: double transgenic R26Fucci2a/CerI-GFP E5.5 embryos were recovered and live imaged. The R26Fucci2a cell cycle reporter was used to identify cells in S/G2/M phase (labelled with mVenus-hGem(301/120) and termed mVenus^+^ cells) and AVE migration was evaluated as distance between leading CerI-GFP^+^ cell and distal tip of the embryo over time. (B) Anterior and posterior view (upper and middle panels) of R26Fucci2a (mVenus signal is shown as multi-coloured LUT Fire) in VE and migrating AVE cells (bottom panel). Extra-Embryonic/Embryonic boundary is shown as dotted line. (C) mVenus^+^ cells in total (upper panel), anterior (middle panel) and posterior (bottom panel) embryonic VE. Number of cells are shown in individual embryos (thin coloured lines) or as mean (thick black line). For comparing all the embryos (raw values are shown in Figs. S1C–E) we considered time 0 h as the time point before the peak (1 h) of mVenus^+^ cells shown in total emVE (bottom panel). (D) Decrease of mVenus^+^ cells per h in the anterior or posterior emVE during time interval 1 h–5 h. Data are shown as mean ± sem (Unpaired *t*-test, *p = 0.03). (E) AVE migration shown as distance between leading CerI-GFP^+^ cell and distal tip of the embryo over time (1 h time interval). Values are shown in individual embryos (thin coloured lines) or as mean (thick black line). For comparing all the embryos (raw values are shown in Fig. S1F) we considered time 0 h as the time point before the peak (1 h) of mVenus^+^ cells shown in total emVE. (F) AVE migration speed shown as μm/h before and after the peak of mVenus^+^ cells. Data are shown as mean ± sem (Unpaired *t*-test, *p = 0.03).

**Fig. 2:**
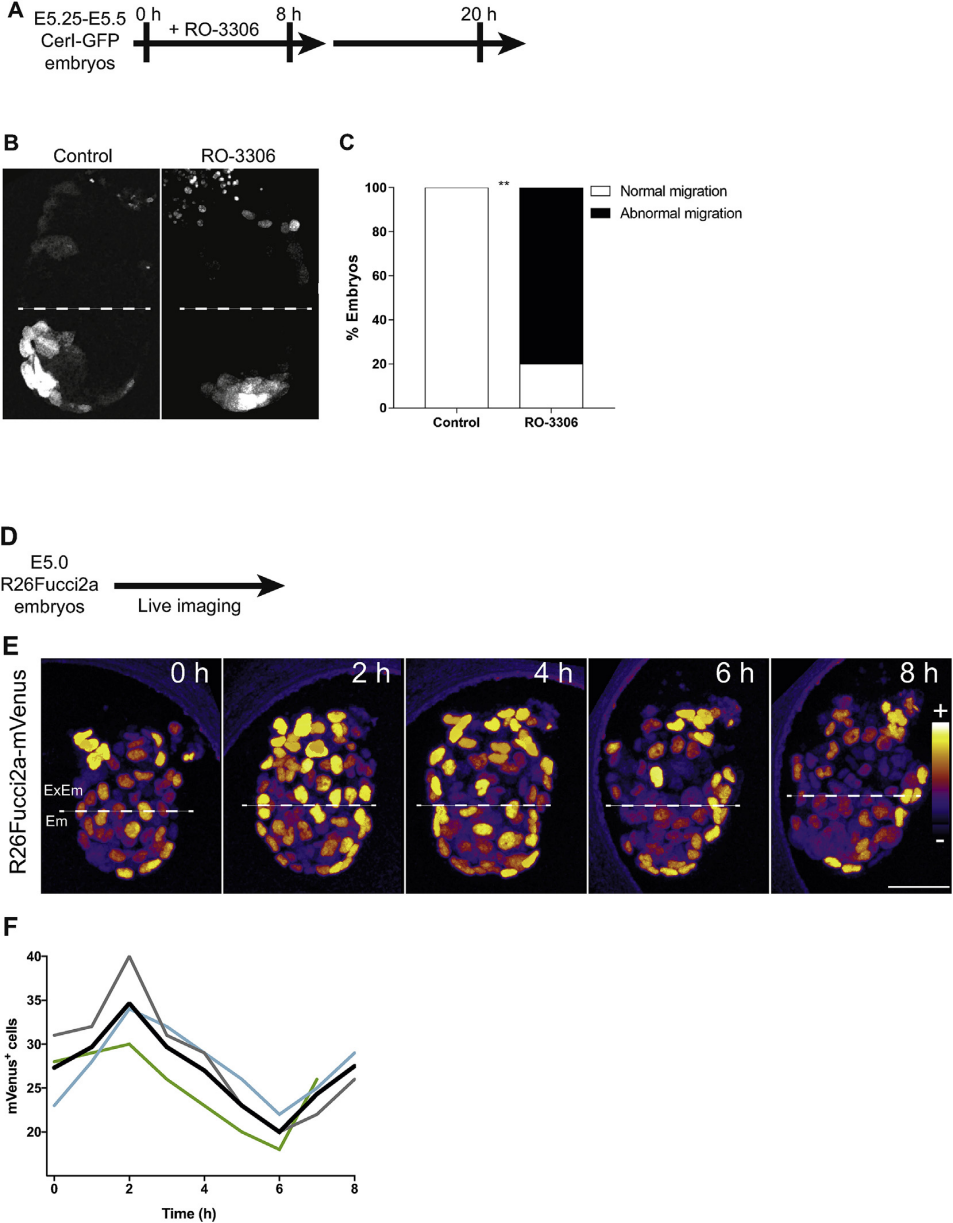
**Coordinated cell divisions are set already at implantation and required during AVE migration**. (A) Schematic view of the experimental design: Cerl-GFP embryos were recovered at E5.25-E5.5 prior AVE migration, cultured in IVC2 medium supplemented with the reversible G2/M inhibitor RO-3306 for 8 h then live-imaged after drug withdrawal. (B) Migrated AVE in control or RO3306-treated embryos 12 h after drug withdrawal. (C) Percentage of embryos showing normal or abnormal AVE migration in control (*n* = 5) or RO3306-treated (n = 5) embryos 12 h after drug withdrawal. Embryos were categorised as normal when AVE migrated until Em/Ex boundary. Otherwise considered abnormal (χ^2^ test, **p = 0.0098). (D) Schematic view of the experimental design: R26Fucci2a embryos were recovered at E5.0 and live imaged. (E) Snapshots of an imaged E5.0 R26Fucci2a (mVenus signal shown as multi-coloured LUT Fire). (F) mVenus^+^ cells in embryonic VE. Number of cells are shown in individual embryos (thin coloured lines) or as mean (thick black line). For comparing all the embryos (raw values are shown in Fig. S2C) we considered time 0 h as the time point before the peak (1 h) of mVenus^+^ cells shown in total emVE.

To enable comparisons between embryos, we determined the number of mVenus^+^ cells in posterior and anterior embryonic parts of VE (emVE) of E5.5 embryos over time (Figs. S1C–E) and normalised temporal progression relative to the time point with the highest number of mVenus^+^ cells. This revealed that within 4 h there was a 36.5% (*n* = 5) decrease in the number of mVenus^+^ cells in the entire emVE indicative of a coordinated burst of cell division (Fig. 1C, upper graph). When we analysed anterior and posterior emVE separately (Fig. 1C middle and lower graphs), the decrease in the number of mVenus^+^ cells appeared more striking in the posterior than in the anterior part of the embryo (49.9% vs 19.6% respectively, *n* = 5) suggesting an element of coordinated cell division in the posterior emVE in a specific time window. We also found a 3-fold difference between the rate of decrease of mVenus^+^ cells in the anterior versus the posterior (Fig. 1D). We measured AVE migration by measuring the distance between the leading CerI-GFP^+^ migratory cell and the distal tip (Fig. 1A) over the same interval (Fig. 1E and Fig. S1F; method for alignment of maximal value indicated in Fig. S1F). We found that the AVE migration speed was significantly higher after cells divided in posterior (2.2 μm/h ± 0.7 μm/h before the peak of mVenus^+^ cells; 5.7 μm/h ± 1.3 μm/h after the peak) (Fig. 1F). These results indicate a spatio-temporal correlation between the timing of AVE migration and cell division in emVE suggesting that coordinated cell cycle progression may contribute to drive AVE migration.

To directly address whether AVE migration is dependent on coordinated cell cycle progression, we recovered Cerl-GFP embryos from mothers prior to AVE specification at E5.25-E5.5 and cultured them in the presence of the reversible Cyclin-Dependent Kinase 1 inhibitor, RO-3306, that arrests cells at G2/M (Jang et al., 2014; Vassilev et al., 2006). We chose to use a Cyclin-Dependent Kinase 1 inhibitor to arrest cells at G2/M instead of a microtubule depolymerising agent, such as Nocodazole, because of the known requirement for microtubules for cell migration (Baudoin et al., 2008). Following 8 h of RO-3306 treatment, we observed a reduction in the number of Gata4 positive emVE and Oct4 positive epiblast cells (Fig. S2 A-C) confirming the efficacy of the drug. Afterwards, we washed out the inhibitor and performed a 12 h live imaging (Fig. 2A). We verified that there was no difference in size between control and RO-3306 treated embryos at the end of the experiment (Figs. S2D–E) suggesting the growth of the embryos was not affected after treatment with the drug. We also found that AVE migration was strongly reduced following RO-3306 treatment (Fig. 2B). Indeed, 85.7% of treated embryos showed abnormal migration in which the Cerl-GFP expressing cells remained in a predominantly distal position (Fig. 2C).

To ask whether the coordinated cell divisions may already take place before AVE specification occurs, we carried out live imaging of early post-implantation E5.0 R26Fucci2a embryos (Fig. 2D–E) and analysed the number of mVenus^+^ cells (Fig. S2F). We observed periodic fluctuations in the number of mVenus^+^ cells (Fig. 2F) and, similar to E5.5 R26Fucci2a/CerI-GFP embryos (Fig. 1C), the fall in the number of mVenus^+^ cells took place over approximately 4 h, suggesting coordinated cell division took place in the emVE immediately following implantation. Together, these results suggest that coordinated cell division contributes to the migration of the AVE during early post-implantation development.

### 2.2 Perturbation of FGF signalling during PE specification affects cell cycle progression in post-implanting embryos

We next wished to identify at which point in development cell cycle control might be imposed upon the developing emVE. We therefore asked whether this might begin already before implantation when the PE is first specified in response to fibroblast growth factor (FGF) signalling (Kang et al., 2017; Molotkov et al., 2017; Morris et al., 2013; Yamanaka et al., 2010). This seemed possible because FGF has also been proposed to act as a mitogen (Ornitz and Itoh, 2015; Turner and Grose, 2010). As growth factor stimulation generally drives cells from a quiescent phase into S phase, we determined the effect of modulating FGF signalling during PE specification by treating E3.5 embryos with the FGFR inhibitor SU5402 for 5 h and assessing S-phase entry by following 5-ethynyl-2^’^-deoxyuridine (EdU) incorporation (Salic and Mitchison, 2008) and mitosis by Phospho-Histone 3 (pH3) immunostaining, using Sox17 as a primitive endoderm marker (Fig. 3A). Before the treatment, the majority of Sox17^+^ cells were in S-phase (83.23%) and no mitoses were detected in the newly specified PE (Fig. 3B). After 5 h, control embryos showed a burst of mitoses in around 9.3% of Sox17^+^ cells and this was reduced to 2.1% following FGF inhibition by the 5 h treatment with SU5402 (Fig. 3C). Nevertheless, in contrast to a longer treatment with FGFR or MEK inhibitors (Krawchuk et al., 2013; Yamanaka et al., 2010), this 5 h pulse of SU5402 did not affect lineage allocation following a 19 h recovery period (Fig. 3D–E).

**Fig. 3:**
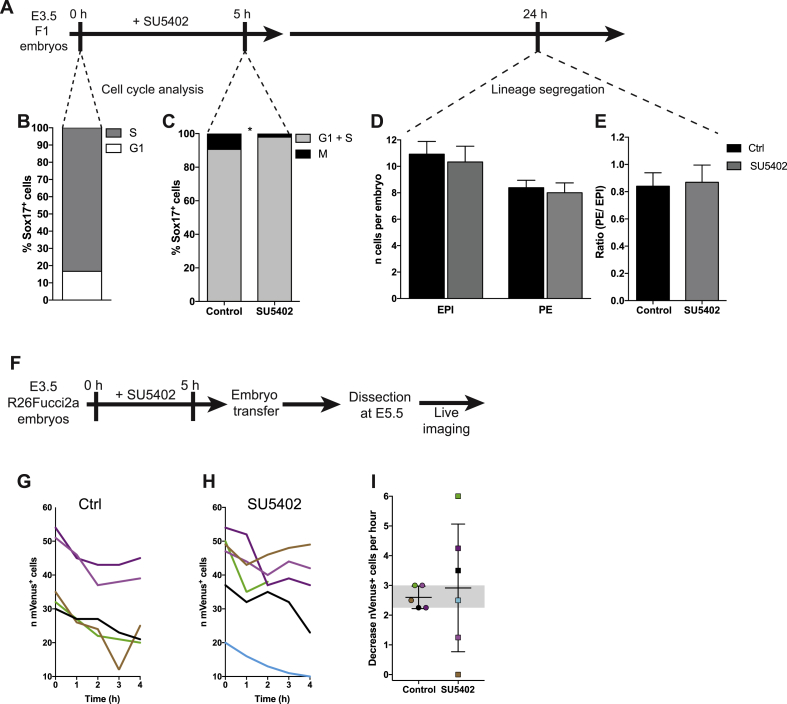
**Perturbation of FGF signalling during PE specification affects cell cycle** (A) Schematic view of the experimental design: embryos were recovered at E3.5 and treated with SU5402 for 5 h and then released in KSOM medium without the drug. EdU labelling and immunostaining for pH3 for assaying cell cycle was performed at 0 h and 5 h while lineage analysis was performed at 24 h. (B) Distribution of G1 and S phases within SOX17^+^ cells at 0 h (*n = *251 cells; 54 embryos). (C) Distribution of G1+S and M phases within SOX17^+^ cells for control (*n = *246; 34 embryos) or SU5402-treated embryos (*n* = 96; 24 embryos) (χ^2^ test, *p = 0.0204). (D) Histograms show the number of Epiblast (Nanog^+^) and PE (Gata4^+^) cells in control or SU5402-treated embryos (control *n* = 13, SU5402 *n = *9). Unpaired *t*-test, ns (Epiblast) or ns (PE). (E) Histograms show the ratio PE/EPI. Unpaired *t*-test, ns. (F) Schematic view of the experimental design: E3.5 R26Fucci2a embryos were treated with SU5402 for 5 h and then transferred to foster mothers. Embryos were recovered at E5.5 and live imaged. (G–H) Plot of mVenus^+^ cells in emVE of control (G) or SU5402-treated (H) embryos in the 4 h following the highest number of cells shown in Figs. S3C–D. Number of mVenus^+^ cells are shown in individual embryos (each colour refers to an embryo and the same colour code is used in panel I). (I) Decrease of mVenus^+^ cells per h in control or SU5402-treated embryos. Data are shown as mean ± sd and the grey band represents the interdecile range (ID) calculated on the control values: 10% Percentile = 2.25and 90% Percentile = 3.

To address the potential effects of the 5 h SU5402 treatment upon development of the VE, we treated E3.5 R26Fucci2a embryos with SU5402 for 5 h, transferred them to foster mothers and subsequently recovered them at E5.5 for analysis by live imaging (Fig. 3F). Both groups of embryos were indistinguishable in size (Figs. S3A–B) indicating that the pre-implantation treatment and subsequent embryo transfer did not lead to differential growth. We then counted the number of mVenus^+^ cells over time in control and SU5402-treated embryos (Figs. S3C–D) focussing on the 4 h interval we had earlier defined in which the numbers of mVenus^+^ cells dramatically decreased corresponding with the time of AVE migration (Fig. 3G–H). Although all embryos showed a decreased number of mVenus^+^ cells, the SU5402-treated embryos showed much higher variability in this rate of decrease (Fig. 3I). These results indicate that short perturbation of FGF signalling affects cell cycle progression during PE specification and has a subsequent impact on the spatio-temporal coordination of cell division at post-implantation stages.

### 2.3 FGF signalling-mediated perturbation of the cell cycle affects post-implantation morphogenesis

**Fig. 4:**
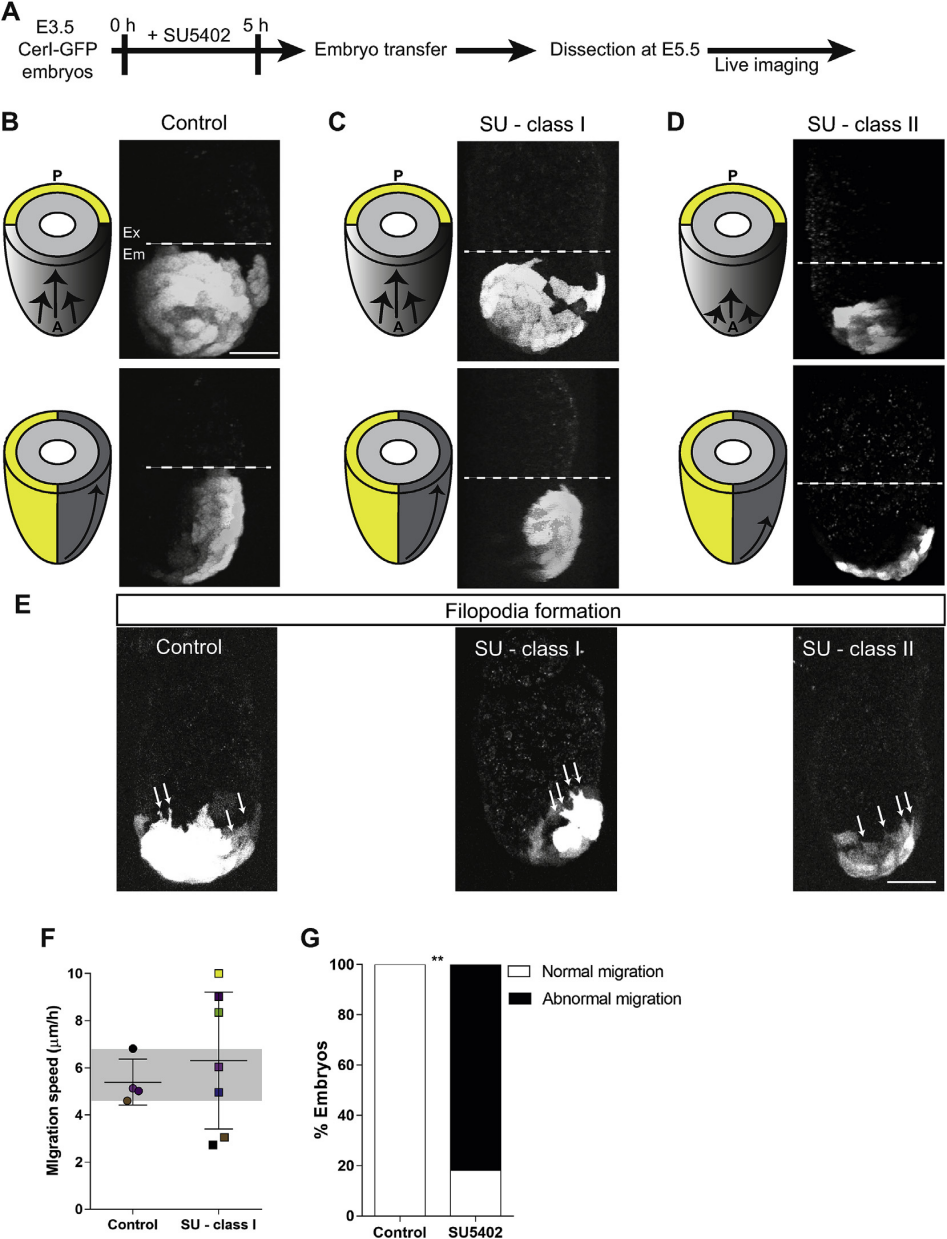
**FGF signalling-mediated perturbation of cell cycle affects post-implantation morphogenesis** (A) Schematic view of the experimental design: E3.5 Cerl-GFP embryos were treated with SU5402 for 5 h and then transferred to foster mothers. Embryos were recovered at E5.5 and live imaged. (B–D) Anterior view (top panels) and lateral view (bottom panels) of migrated AVE in Cerl-GFP embryos. Embryos were divided based on the phenotype in control (B) and SU5402 treated embryos class I (SU - class I) (C) or class II (SU - class II). (D) (Scale bar = 50 μm). (E) Filopodia in migrating CerI-GFP^+^ cells showed in control (left panel) and SU5402 treated embryos class I (middle panel) or class II (left panel). Arrows indicate filopodia. (Scale bar = 50 μm). (F) AVE migration speed shown as μm/h in control or SU – class I embryos. Data are shown as mean ± sd (Unpaired *t*-test, ns). The grey band represents the interdecile range (ID) calculated on the control values: 10% Percentile = 4.6 and 90% Percentile = 6.8. (G) Percentage of control or SU5402-treated embryos (class I and class II) showing normal or abnormal AVE migration in control (*n* = 4) or SU5402-treated embryos (*n* = 11) (χ^2^ test, **p = 0.0042).

In the light of these results, we asked whether perturbing FGF signalling already at the blastocyst stage would affect AVE migration. To this end, we treated Cerl-GFP E3.5 embryos for 5 h with SU5402, transferred them to foster mothers and recovered them at E5.5 and assessed the directionality and speed of AVE migration (Fig. 4A). As expected, in 100% of control embryos, AVE cells migrated unidirectionally from the distal tip towards the ExE/EPI boundary (Fig. 4B, *n* = 4). However, amongst the SU5402-treated embryos only 63.6% (*n* = 11) of embryos displayed unilateral migration similar to controls (SU5402-treated - Class I; Fig. 4C). By contrast, the remaining 36.4% embryos (SU5402-treated - Class II) showed aberrant migration, with AVE cells not fully reaching the Em/Ex boundary (Fig. 4D). Despite the different phenotype between control and SU5402-treated embryos, there was no difference in their size at the time of the recovery (Figs. S4A–B). In addition, all groups exhibited formation of filopodia indicating that the short treatment with FGFR inhibitor performed 3 days earlier did not affect the active cell migration machinery (Fig. 4D). Despite the apparent unaffected migration path, we investigated whether SU5402 - class I showed any difference in AVE migration speed. To this end, we assessed AVE migration by measuring the distance between the leading CerI-GFP^+^ migratory cell and the distal tip over time (Figs. S4C–D). We found that whereas the AVE of control embryos displayed a migration speed of 5.4 μm/h ± 0.5 μm/h, the AVE of SU5402-treated - Class I embryos migrated slightly faster (6.3 μm/h ± 1.1 μm/h) and with a higher variability (Fig. 4F). When we considered the AVE migration speed of individual SU5402-treated - Class I embryos, we observed that 5/7 resided outside of the control interdecile range (ID range shown in grey) (Fig. 4F). Interestingly, despite unidirectional migration of AVE, SU5402-treated - Class I embryos displayed significant alterations in AVE migration, which, taken together with the aberrant AVE migration in SU5402-treated – Class II embryos, indicate that 81.8% of the embryos had abnormal AVE migration (Fig. 4G).

Given that inhibition of FGF signalling during pre-implantation resulted in abnormal AVE migration, we next asked whether cell fate, AP patterning and later post-implantation development were also affected. We found that at E6.5, SU5402-treated embryos stained for Brachyury, an early mesodermal lineage marker expressed in the primitive streak, were indistinguishable from control embryos (Figs. S4E–F) with both groups showing unilateral formation of the primitive streak in all embryos (*n* = 4 control and *n* = 6 SU5402-treated embryos). In addition, both groups showed formation of basement membrane (Fig. S4F, bottom panels) that is known to be directly controlled by FGF (Costello et al., 2009). This suggests that treatment with SU5402 acted specifically on cell cycle/AVE migration.

Together our findings demonstrate that the migration of AVE cells is coordinated with and dependent upon the progression of VE cells through the cell division cycle.

## 3 Discussion

Mouse embryos undergo morphogenetic remodelling at the time of implantation during which the VE shapes the AP patterning of the EPI (Brennan et al., 2001). This process is mediated by a specialised population of VE cells, which following their specification at the distal tip of the embryo, migrate towards the future anterior side (Morris et al., 2012). This is a highly coordinated process requiring AVE cells to actively migrate (Migeotte et al., 2010; Morris et al., 2012; Omelchenko et al., 2014; Srinivas et al., 2004) as the entire emVE undergoes rearrangement in cell shape (Stower and Srinivas, 2014) whereas cell proliferation leads the embryo to increase in size. We sought to address whether coordinated progression through the cell cycle is required for AVE migration. To gain spatio-temporal resolution of cell division, we carried out live imaging. This revealed that cell divisions are coordinated in the emVE compartment during and before AVE migration and that AVE migration drastically increases in speed as cells divide. Moreover, we find that proper migration of AVE is impaired when cell cycle progression is transiently perturbed. In embryos exposed to the G2/M reversible inhibitor RO-3306, the AVE failed to migrate correctly even after release into fresh medium. It has previously been proposed that AVE migration could be driven by differential proliferation within emVE resulting from asymmetry in Nodal signalling (Yamamoto et al., 2004). In contrast, more recent reports failed to detect significant differences in proliferation amongst different regions of the emVE (Shioi et al., 2017; Stuckey et al., 2011a). However, these studies did not analyse the dynamics of mitoses in the emVE at the time of AVE migration. Here by using transgenic lines reporting both the AVE and specific cell cycle stages, we have been able to show that cell migration speed changes following a coordinated wave of cell divisions in the posterior side of the embryo, opposite to the site of AVE migration. We speculate that bursts of cell division, in concert with the complex remodelling of the whole emVE due to cell intercalation driven by PCP signalling (Trichas et al., 2012), generates sufficient force to enable the AVE to migrate.

Our live imaging of peri-implantation embryos also revealed that coordinated cell divisions are already present in the VE shortly after the embryo implants. While our results clearly demonstrate that coordinated mitoses are required for the correct migration of the AVE, the cellular underpinnings of this phenomenon remain to be fully characterised. Since we observed coordinated mitotic events *ex vivo* shortly after implantation it is tempting to speculate that regulated cell divisions have their origins before implantation at the time of PE specification. This hypothesis is strengthened by the observation that a short perturbation of FGF signalling does not affect lineage commitment but does alter cell cycle progression in PE cells following transfer to foster mothers.

In addition to its role in the EPI/PE fate decision (Kang et al., 2017; Molotkov et al., 2017; Morris et al., 2013; Yamanaka et al., 2010), the FGF signalling pathway has been described to regulate cell proliferation or cell cycle arrest in a context-dependent manner (Ornitz and Itoh, 2015; Turner and Grose, 2010). FGF has been shown to act via both FGFR1 and FGFR2 (Kang et al., 2017; Molotkov et al., 2017) and hypothesised to control proliferation and survival of the PE (Molotkov et al., 2017). Our findings of a decrease in the number of mitotic PE cells after FGFRs inhibition are in agreement with a proliferative role of FGF signalling during pre-implantation development (Fig. 3C). The impact of FGFR inhibition on cell cycle progression was also observed when embryos were transferred back to the mother and recovered at E5.5 (Fig. 3G–I). Strikingly, a pulse of FGFR inhibition in the blastocyst affected the speed (Fig. 4F) and direction of AVE migration (Fig. 4B–D), even though CerI-GFP^+^ cells had a morphology typical of cells able to be actively involved in migration (Fig. 4E).

Given the limitations of working with the mouse embryo system, it is difficult to pinpoint the exact mechanisms underpinning cell cycle coordination in PE precursors. One possibility is that cell-to-cell communication may be involved. Cell-to-cell communication plays an important role in variety of biological phenomena, including cell migration and lineage specification. In mouse development, communication between PE and EPI progenitors determines their specification and relies on FGF signalling (Kang et al., 2017; Molotkov et al., 2017). We surmise that the progeny of PE cells is able to maintain previously acquired coordination in cell cycle during their differentiation into AVE. This does not exclude the contribution of cell-to-cell communication to AVE migration, possibly in a cell cycle independent fashion. It has been recently shown that exchange of information between cells via molecular diffusion and transport processes helps guide their concerted movement in the presence of external chemical cues during mammary gland development (Ellison et al., 2016). Since regionalisation of AVE cells to the anterior side of mouse embryos relies on a gradient of Nodal signalling (Yamamoto et al., 2004), it is possible that a similar mechanism could also be at play during AVE migration in mouse embryos. However, it is unclear whether the contribution of intercellular interactions may be accompanied by or mediated by changes in cell cycle in migrating cells.

The AVE has a pivotal role in the positioning of primitive streak (Stuckey et al., 2011b). Indeed, genetic mutations in signalling pathways or apical cell polarity affecting AVE migration display defects in primitive streak positioning or expansion (Stower and Srinivas, 2014). In this study, we report that short pharmacological perturbation of FGF signalling by disrupting cell cycle coordination in the VE selectively impairs AVE migration but does not affect cell fate or primitive streak formation. This discrepancy could be explained by the fact that following SU5402 treatment, despite their aberrant migration, AVE cells primarily resided on the anterior side of the embryo, thus enabling correct positioning of the primitive streak. Moreover, as we observed formation of primitive streak and basement membrane deposition in SU5402 treated embryos (Fig. S4F), the signalling pathways involved in these processes, such as FGF, Nodal, Wnt and TGFb (Costello et al., 2009; Tam and Behringer, 1997), were most likely unaffected by transient FGF inhibition. Therefore, we postulate that the long-term consequences of SU5402 treatment may be cell-cycle specific. In addition to its effect on cell division, we cannot exclude that inhibition of FGF signalling may affect cell migration directly, as FGFs have been previously shown to act as chemoattractant (Bae et al., 2012; Kubota and Ito, 2000). Although it is difficult to rule out this possibility, the fact that Brachyury^+^ cells were specified and underwent migration in treated embryos, as previously discussed, seems to suggest that FGF signalling was functional post-implantation and that FGFR inhibition had its impact primarily on cell division.

Taken together, our findings reveal that FGF signalling, known to be involved in EPI/PE segregation, also facilitates coordination of the cell cycle within PE progenitors. Moreover, we have demonstrated that coordinated cell division contributes to tissue remodelling and cell movements necessary for AVE migration. To our knowledge, this is the first study showing the requirement for FGF-mediated coordinated cell cycle progression in PE cells for proper AVE migration and for morphogenetic events in mouse post-implantation embryos.

## 4 Materials and methods

### 4.1 Animals

This research has been regulated under the Animals (Scientific Procedures) Act 1986 Amendment Regulations 2012 following ethical review by the University of Cambridge Animal Welfare and Ethical Review Body (AWERB). The following mouse lines were used for the study: R26Fucci2a (Mort et al., 2014), Cerl-GFP (Mesnard et al., 2004), F1 (C57BL/6 × CBA).

### 4.2 Embryo recovery and culture

Pre-implantation embryos (E3.5 – E4.75) were flushed out from the uteri as described in Bedzhov et al., 2014). E3.5 embryos were cultured in drops of KSOM (Millipore, MR-020P) supplemented with SU-5402 10 μM (Abcam, ab146602) for 5 h and then KSOM only for 19 h. Control embryos were cultured in KSOM supplemented with 0.1% DMSO (Sigma, D2650). Post-implantation embryos (E5.0 - E5.25 - E5.5) were dissected at room temperature in M2 medium and then transferred in IVC2 medium (Bedzhov et al., 2014). Pharmacological perturbation of cell cycle was performed culturing early E5.5 embryos in IVC2 supplemented with the G2/M inhibitor RO-3306 10 μM (Sigma, SML0569). Embryos were live imaged on glass bottom dishes (MatTek Corporation, P35G-1.5-14-C). Embryos were cultured at 37 °C in 5% CO_2_.

### 4.3 Imaging

Embryos were live imaged using a Leica SP8 Multi-photon confocal microscope. The wavelengths for confocal excitation were 514 nm laser (for mVenus of R26Fucci2a), 488 nm (for CerI-GFP) while for multi-photon excitation 1040 nm was used for R26Fucci2a embryos (when double-transgenic embryos R26Fucci2a/CerI-GFP embryos imaged). Fixed embryos were imaged on either Leica SP5 or SP8 confocal microscopes.

### 4.4 Immunostaining and EdU labelling

Embryos were fixed in 4% paraformaldehyde for 20 min at room temperature and permeabilized with 0.3% Triton X-100/0.1 M Glycine in PBS for 20 (pre-implantation) or 30 (post-implantation) min at room temperature. The primary antibodies were added to the Antibody solution (0.1% Tween-20/10% filtered FCS in PBS) and samples were incubated at 4 °C overnight. Embryos were then incubated for 2 h at room temperature with secondary antibodies in Antibody solution. All washes were done in PBST (PBS + 0.1% Tween-20). DAPI was used as a nuclear counterstain. EdU labelling was performed before fixation by culturing E3.5 embryos in KSOM supplemented with EdU 10 μM for 20 min. EdU detection was performed in according to manufacturer instructions (Thermo Fisher Scientific, C10339). Primary antibodies used: anti-Sox17 (R&D system, af1924), anti-Gata4, anti-Nanog (Abcam, ab80892), anti-Oct4 (Santa Cruz, sc-5279), anti-phospho-Histone-H3 (Millipore, 06-570), anti-Brachyury (R&D system, AF2085).

### 4.5 Image processing and statistical analysis

Fiji software was used for image processing and Graphpad prism was used for graph design and statistical analysis.

## Conflicts of interest

The authors declare no competing or financial interests.

## Author contribution

F.A. designed, performed and analysed all the experiments. L.C.O. helped with some experiments and analysis of data. R.L.M. provided the R26Fucci2a mouse line. F.A. and M.Z.G. conceived the project and wrote the manuscript.

## Funding

This work was supported by Wellcome Trust (098287/Z/12/Z) and European Research Council (669198) grants to M.Z.G., F.A. was supported by an EMBO postdoctoral fellowship and L.C.O. was supported by a Centre for Trophoblast Research PhD studentship.

## Figures and Tables

**figs1:**
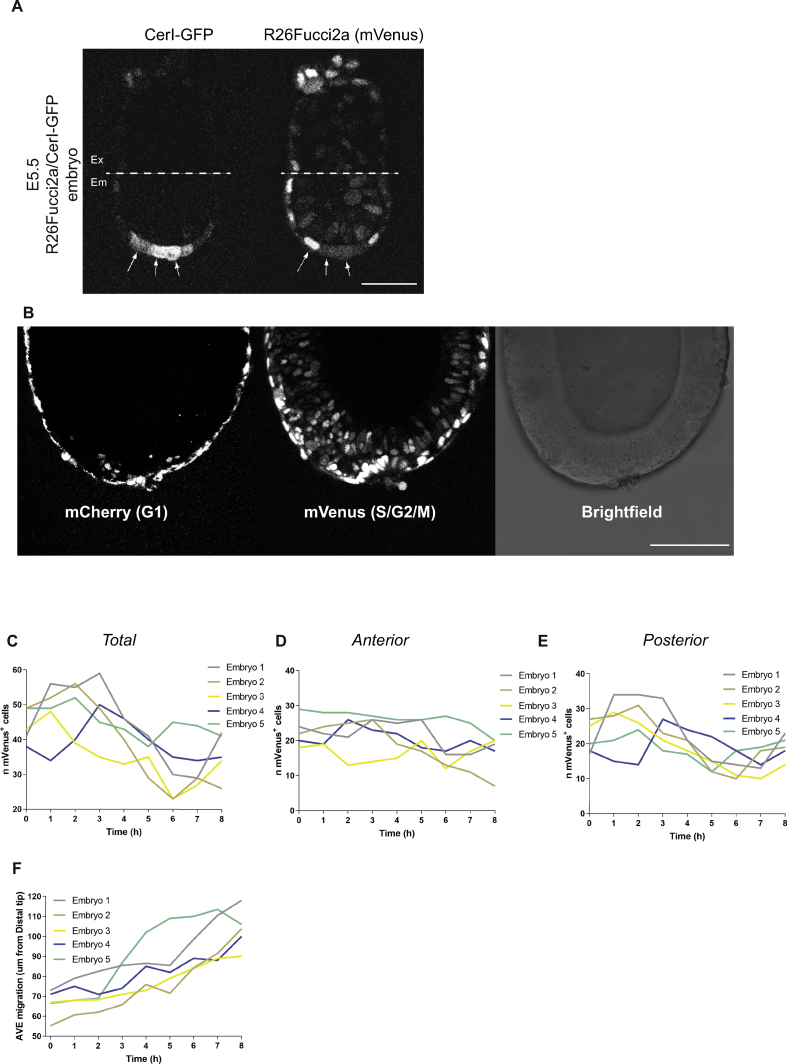


**figs2:**
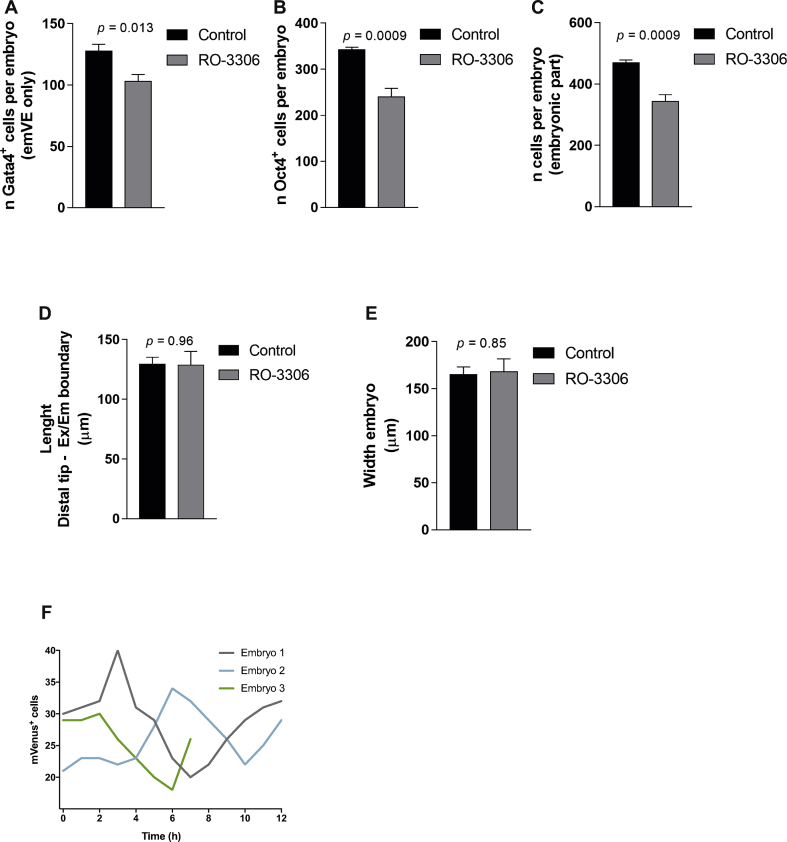


**figs3:**
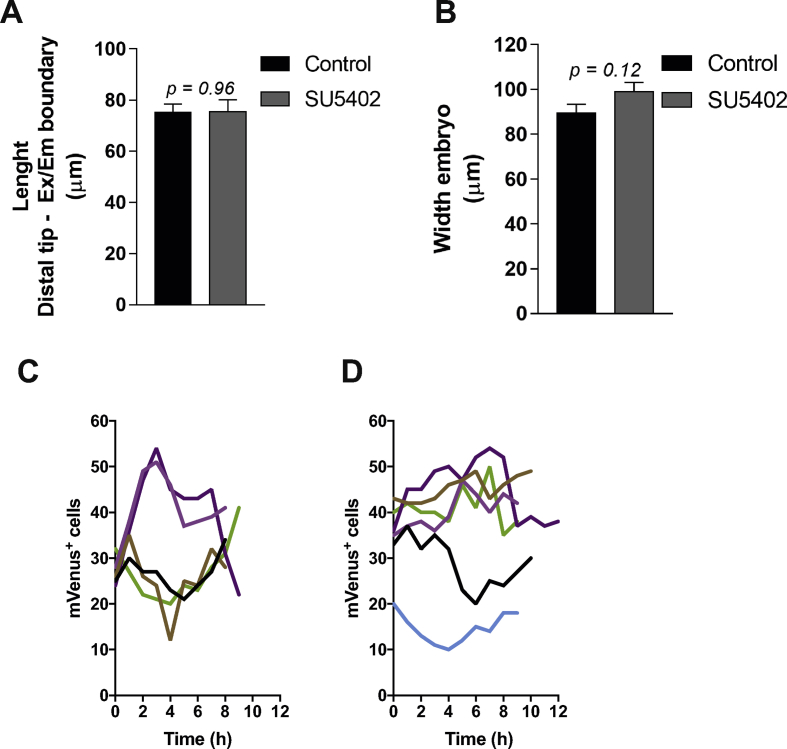


**figs4:**